# Comparison of two preoperative chemoradiotherapy regimens for locally advanced rectal cancer: capecitabine alone *versus* capecitabine plus irinotecan

**DOI:** 10.1186/1748-717X-8-258

**Published:** 2013-11-04

**Authors:** Sung Uk Lee, Dae Yong Kim, Sun Young Kim, Ji Yeon Baek, Hee Jin Chang, Min Ju Kim, Tae Hyun Kim, Ji Won Park, Jae Hwan Oh

**Affiliations:** 1 Proton Therapy Center, Goyang, Republic of Korea; 2 Center for Colorectal Cancer, Research Institute and Hospital, National Cancer Center, Goyang, Republic of Korea

**Keywords:** Rectal cancer, Preoperative chemoradiotherapy, Capecitabine, Irinotecan

## Abstract

**Background:**

To compare the short-term tumor response and long-term clinical outcome of two preoperative chemoradiotherapy (CRT) regimens for locally advanced rectal cancer.

**Methods:**

This study included 231 patients scheduled for preoperative CRT using two chemotherapeutic protocols from April 2003–August 2006. Pelvic radiotherapy (50.4 Gy) was delivered concurrently with capecitabine (*n* = 148) or capecitabine/irinotecan (*n =* 83). Surgery was performed 4–8 weeks after CRT completion. Tumor responses to CRT were assessed using both radiologic and pathologic measurements. Radiologic responses were evaluated by magnetic resonance volumetry, which was performed at the initial work-up and after completion of preoperative CRT just before surgery. Pathologic responses were assessed with downstaging (ypStage 0-1) and grading tumor regression. Clinical outcomes were evaluated in terms of local control, relapse-free survival, and overall survival rates.

**Results:**

Radiologic examination demonstrated that tumor volume decreased by 65.6% in the capecitabine group and 66.8% capecitabine/irinotecan group (*p* = 0.731). Postoperative pathologic stage determination showed that tumor downstaging occurred in 44.1% of the capecitabine group and 48.6% of the capecitabine/irinotecan group (*p* = 0.538). The sum of tumor regression grade 3 (near complete response) and 4 (complete response) after CRT were 28.6% in the capecitabine group and 37.5% in the capecitabine/irinotecan group (*p* = 0.247). There were no significant differences between the two groups in 5-year local control (91.7% *vs.* 92.5%; *p* = 0.875), relapse-free survival (80.8% *vs.* 77.2%; *p* = 0.685), and overall survival (88.4% *vs.* 90.4%; *p* = 0.723).

**Conclusions:**

This study revealed no differences in the short-term tumor response and long-term clinical outcome between preoperative capecitabine and capecitabine/irinotecan CRT regimens for locally advanced rectal cancer.

## Background

Locally advanced rectal cancer is currently treated with a multidisciplinary approach since the combination of surgery, chemotherapy and radiotherapy is necessary for an optimal outcome. Preoperative chemoradiotherapy (CRT) followed by radical surgery has become one of the standard treatments for patients with locally advanced rectal cancer and is superior to postoperative CRT by reducing toxicity and improving the local control rate [1]. The most commonly used chemotherapeutic agents for CRT are fluoropyrimidines, including 5-fluorouracil and capecitabine. Although preoperative CRT with 5-fluorouracil has become part of standard therapy in patients with locally advanced rectal cancer, the oral fluoropyrimidine capecitabine has often be used in place of 5-fluorouracil in CRT because of its convenience and safety profile [2]. In addition, capecitabine is highly selective toward tumor tissue because thymidine phosphorylase, essential for capecitabine activation, is more abundantly expressed in tumors than in normal tissue [3]. A recent multicenter phase III trial in Germany confirmed that the endpoint overall survival rates did not change when infused 5-fluorouracil was replaced by the oral prodrug capecitabine during radiotherapy and adjuvant chemotherapy [4].

Irinotecan, a topoisomerase I inhibitor, has shown efficacy in combination with fluoropyrimidines in metastatic colorectal cancer [5]. Several perioperative CRT trials have demonstrated the feasibility of irinotecan and capecitabine [6], and more recently, we reported the clinical outcomes of the drugs in a phase II trial [7]. Although preoperative CRT using the capecitabine/irinotecan regimen has proven effective, those studies did not directly compare capecitabine alone and capecitabine/irinotecan as preoperative CRT regimens for rectal cancer.

The aim of this study was to determine whether the combination of irinotecan with capecitabine could be more effective than capecitabine alone. Here, we report our experience with capecitabine *vs.* capecitabine/irinotecan in evaluating the tumor response and survival after preoperative CRT for locally advanced rectal cancer when the chemotherapeutic agents were administered in conjunction with preoperative radiotherapy.

## Methods

### Patients

The medical records of rectal adenocarcinoma patients from April 2003–August 2006 were retrospectively reviewed. Before April 2003, preoperative CRT with 5- fluorouracil had been mainly used, however during this period of time, the capecitabine started to be used as preoperative CRT regimen and capecitabine/irinotecan protocol was executed. This study included 231 patients with primary rectal cancer who underwent preoperative CRT under two chemotherapeutic regimens, either capecitabine or capecitabine/irinotecan. The inclusion criteria were: *(1)* histologically proven adenocarcinoma (<;9 cm above the anal verge); *(2)* locally advanced disease clinically staged as T3–4 and curatively resectable, evaluated by pelvic magnetic resonance imaging with or without transrectal ultrasonography; *(3)* no evidence of distant metastasis in staging work-up.

Among the 231 patients, six refused surgery (only in capecitabine group) and five patients in the capecitabine group and eight patients in the capecitabine/irinotecan group were treated with transanal local excision respectively, because their co-morbidities made radical proctectomy impossible or they strongly disagreed with curative surgery. In addition, three patients in capecitabine alone group and one patient in the capecitabine/irinotecan group moved to other hospitals to be closer to their residence before surgery. The study was performed in accordance with the guidelines of our institutional review board, which deemed that informed consent was not required because the study was a retrospective analysis.

### Treatment

#### Preoperative chemotherapy

Preoperative chemotherapy was delivered concurrently with pelvic radiation in 150 patients receiving capecitabine alone and 81 receiving capecitabine/irinotecan. The capecitabine-only group was administered an oral 825 mg/m^2^ dose twice daily with no drug holiday for the duration of radiotherapy. The capecitabine/irinotecan group received concurrent chemotherapy with 40 mg/m^2^ of irinotecan per week for 5 consecutive weeks and oral capecitabine at an 825 mg/m^2^ dose twice per day (weekdays only) for the duration of radiotherapy. The capecitabine protocol was executed between April 2003–April 2006, and capecitabine/irinotecan protocol between August 2004–August 2006. In the overlapping period, the protocol undertaken was determined according to the preferences of patients or attending physicians.

#### Radiotherapy

Preoperative radiotherapy of 45 Gy/25 fractions was delivered to the whole pelvis, followed by 5.4 Gy/3 fractions boost to a restricted volume. All patients underwent computed tomography (CT) simulation for three-dimensional conformal radiotherapy, and had their target volumes delineated according to the International Commission on Radiation Units and Measurements Report 50.

#### Surgery

Patients underwent open radical surgery 4–8 weeks (median = 6 weeks) after completion of preoperative CRT. Total mesorectal excision was the priority for surgical treatment, with the final decision regarding the choice of surgical procedure (low anterior or abdominoperineal resection) being made by the surgeon after discussion with the multidisciplinary team involved in the patient’s treatment.

#### Postoperative chemotherapy

Regardless of pathologic stage, all patients underwent postoperative chemotherapy, initiated within 3 or 4 weeks after surgery. One of the following chemotherapeutic regimens was used: four cycles of 5-fluorouracil/leucovorin; six cycles of capecitabine; or six cycles of capecitabine and oxaliplatin.

### Evaluation

Short- and long-term endpoints were evaluated. The short-term endpoint compared tumor response between the two groups, and the long-term endpoint compared the survival rates of the two groups.

Short-term tumor responses to CRT were assessed by: *(1)* radiologic evaluation and *(2)* pathologic evaluation. To evaluate the radiologic responses of the tumor, magnetic resonance volumetry was performed at the initial workup and 2–4 days before surgery. Cross-sectional areas of the lesions were measured by tracing the lesion boundary on axial T2-weighted images. Based on magnetic resonance volumetry, the tumor volume reduction rate was calculated using the equation R (%) = (V_preCRT_ – V_postCRT_) × 100/V_preCRT_, where R represents the tumor volume reduction rate, V_preCRT_ represents the pre-CRT tumor volume, and V_postCRT_ represents the post-CRT tumor volume. Clinical response was defined as a volume reduction rate of ≥ 65% [8].

Pathologic responses of tumors were assessed by collecting the following data for each surgical specimen: histological adenocarcinoma grade, ypStage according to the American Joint Committee on Cancer staging system (6th edition) [9], and tumor regression grade. Downstaging was determined by comparing the pretreatment clinical and postoperative pathologic classifications and defined as ypStage 0–I. Tumor regression grade was classified using the scale proposed by Dworak et al [10]. We defined the overall tumor regression as Grade 3 (near complete response) and 4 (complete response) for statistical analysis.

### Patient follow-up

Patient follow-up was performed every 3 months for the first 2 postoperative years and every 6 months thereafter. Chest radiography and CT scanning of the abdomen and pelvis were conducted every 6 months after surgery, and video colonoscopy was performed at 1, 3, and 5 years after surgery. The diagnosis of recurrence was confirmed pathologically by surgical resection, biopsy or cytology, and/or radiologic findings that increased in size over time. Local recurrence was defined as any disease recurrence within the pelvis. Recurrence outside the pelvis was classified as a distant metastasis.

### Statistical analysis

This study was designed to retrospectively compare the efficacy of two protocols by assessing tumor response and survival rate. As mentioned above, radiologic and the pathologic evaluations were used to evaluate tumor response. The radiologic findings were analyzed to determine the mean volume reduction rate and clinical response rate (volume reduction rate ≥ 65%). The pathologic findings were used to determine downstaging and overall tumor regression. The *t*-test and Fisher’s exact test were used to compare various parameters between the two chemotherapy groups. Relapse-free survival analysis was based on the time of disease recurrence, and overall survival analysis was based on the time of death from any cause. Kaplan-Meier analysis was used to construct relapse-free survival and overall survival curves. Differences with *p* <; 0.05 were considered to be statistically significant.

## Results

### Patient characteristics

The study population had a median age of 56 years (range = 31–83 years) and was predominantly male (133 males, 75 females). The median distance from the anal verge to the caudal edge of the tumor was 5 cm (range = 0–9 cm). The clinical staging work-up revealed cT3 in 201 patients (96.6%) and cT4 in seven patients (3.4%). The patients’ characteristics were recorded according to the two chemotherapeutic regimens and are presented in Table 1. Statistical analysis revealed no significant differences between the capecitabine and capecitabine/irinotecan groups in any category.

**Table 1:** Patients’ characteristics

**Characteristic**	**Capecitabine alone (*n*** **= 136)**	**Capecitabine/Irinotecan (*n*** **= 72)**	***p-*** **value**
Gender			0.991*
Male	87 (64.0)	46 (63.9)	
Female	49 (36.0)	26 (36.1)	
Age (years)			0.191†
Median, Range	57, 31-83	55, 32-74	
Distance from anal verge (cm)			0.326†
Median, Range	5.5, 0-9.0	5, 0.5-9.0	
cT classification			0.732*
cT3	131 (96.3)	70 (97.2)	
cT4	5 (3.7)	2 (2.8)	
Pre-CRT CEA (ng/ml)			0.319†
5 ≥	87 (64.0)	51 (70.8)	
5 <;	49 (36.0)	21 (29.2)	

CEA, carcinoembryonic antigen; CRT, chemoradiotherapy.

Data in parentheses are percentages.

*Two-tailed Fisher’s exact tests.

†Two-tailed *t*-test.

### Radiologic findings

Magnetic resonance volumetry assessment was performed for 216 patients (93.5%). The median pre-CRT tumor volumes were 17.5 cm^3^ for capecitabine alone and 16.2 cm^3^ for capecitabine/irinotecan (*p* = 0.625). The median post-CRT tumor volume was 5.6 cm^3^ for capecitabine alone and 5.2 cm^3^ for capecitabine/irinotecan (*p* = 0.681). Of the 216 patients, 134 (62.0%) had clinical responses in which the tumor volume was reduced ≥ 65%. The radiologic findings showed no differences between the capecitabine and capecitabine/irinotecan groups in terms of tumor volume reduction rate and clinical response (Table 2).

**Table 2:** Radiologic and pathologic evaluations of short-term tumor response

	**Short-term tumor response**	**Capecitabine alone**	**Capecitabine/Irinotecan**	***p-*** **value**
Radiologic findings	Patients number	137	79	
	Tumor volume reduction rate† (%)			0.731*
	Mean	65.6 ± 24.3	66.8 ± 22.5	
	Median	71.7	72.0	
	Range	0–100	2.3–100	
	Clinical response (tumor volume reduction rate ≥ 65%)	82 (60)	52 (66)	0.384‡
Pathologic findings	Patients number	131	70	
	Post-CRT stage			0.538‡
	0-I	60 (44.1)	35 (48.6)	
	II-IV	76 (55.9)	37 (51.4)	
	Tumor regression grade			0.247‡
	1 or 2	96 (71.3)	45 (62.5)	
	3 or 4	40 (28.6)	27 (37.5)	

CRT, chemoradiotherapy.

*Two-tailed *t*-test.

†Tumor volume reduction rate = (pre-CRT tumor volume – post-CRT tumor volume) × 100/pre-CRT tumor volume.

‡Two-tailed Fisher’s exact test.

### Surgery

A total of 208 patients underwent curative surgery, although 11 patients in the capecitabine group and seven in the capecitabine/irinotecan group had a positive microscopic circumferential resection margin. Distant metastasis, which was not detected in the initial staging work-ups, was discovered in the liver in only one patient in the capecitabine alone group during surgery. The sphincter was preserved in 116 (85.2%) of 136 capecitabine alone patients and 60 (83.3%) of 72 capecitabine/irinotecan patients (*p* = 0.711). Of the 43 patients whose tumors were located within 3 cm of the anal verge and were likely to require sphincter ablation according to previous data [11], the anal sphincter was preserved in 11 (40.7%) of 27 capecitabine alone patients and six (37.5%) of 16 capecitabine/irinotecan patients (*p* = 0.838).

### Pathologic findings

After radical surgery, pathologic assessment for 208 patients (100%) was undertaken. The pathologic staging of surgical specimens showed ypStage 0 in 37 patients (17.8%), ypStage I in 58 patients (27.9%), ypStage II in 45 patients (21.6%), ypStage III in 67 patients (32.2%), and ypStage IV in 1 (0.5%) patient. As a result of preoperative CRT, downstaging to ypStage 0 or I occurred in 44.1% of capecitabine patients and 48.6% of capecitabine/irinotecan patients; no significant difference between two groups was noted (*p* = 0.538).

Tumor regression in all patients was also evaluated. According to Dworak’s regression grading scale, 32 patients (15.4%) were Grade 1, 109 (52.4%) were Grade 2, 31 (14.9%) were Grade 3, and 36 (17.3%) were Grade 4. Overall tumor regression (including regression at Grades 3 and 4) occurred in 29.4% of capecitabine patients and 37.5% of capecitabine/irinotecan patients and showed no significant differences (*p* = 0.247; Table 2).

### Postoperative chemotherapy

Among the 208 patients who completed curative resection, 129 (94.9%) of 136 capecitabine-alone patients and 72 (100%) of 72 capecitabine/irinotecan patients (*p* = 0.098) started postoperative chemotherapy. Reasons for not continuing with postoperative chemotherapy included postoperative complications (*n* = 1), cytopenia (*n* = 2), patient refusal (*n* = 3) and poor performance status (*n* = 1). The adjuvant chemotherapy regimen in the capecitabine-alone group and capecitabine/irinotecan group was fluoropyrimidine alone (91.9% *vs.* 94.4%) and in combination with oxaliplatin (2.9% *vs*. 5.6%).

### Clinical outcomes

**Figure 1:**
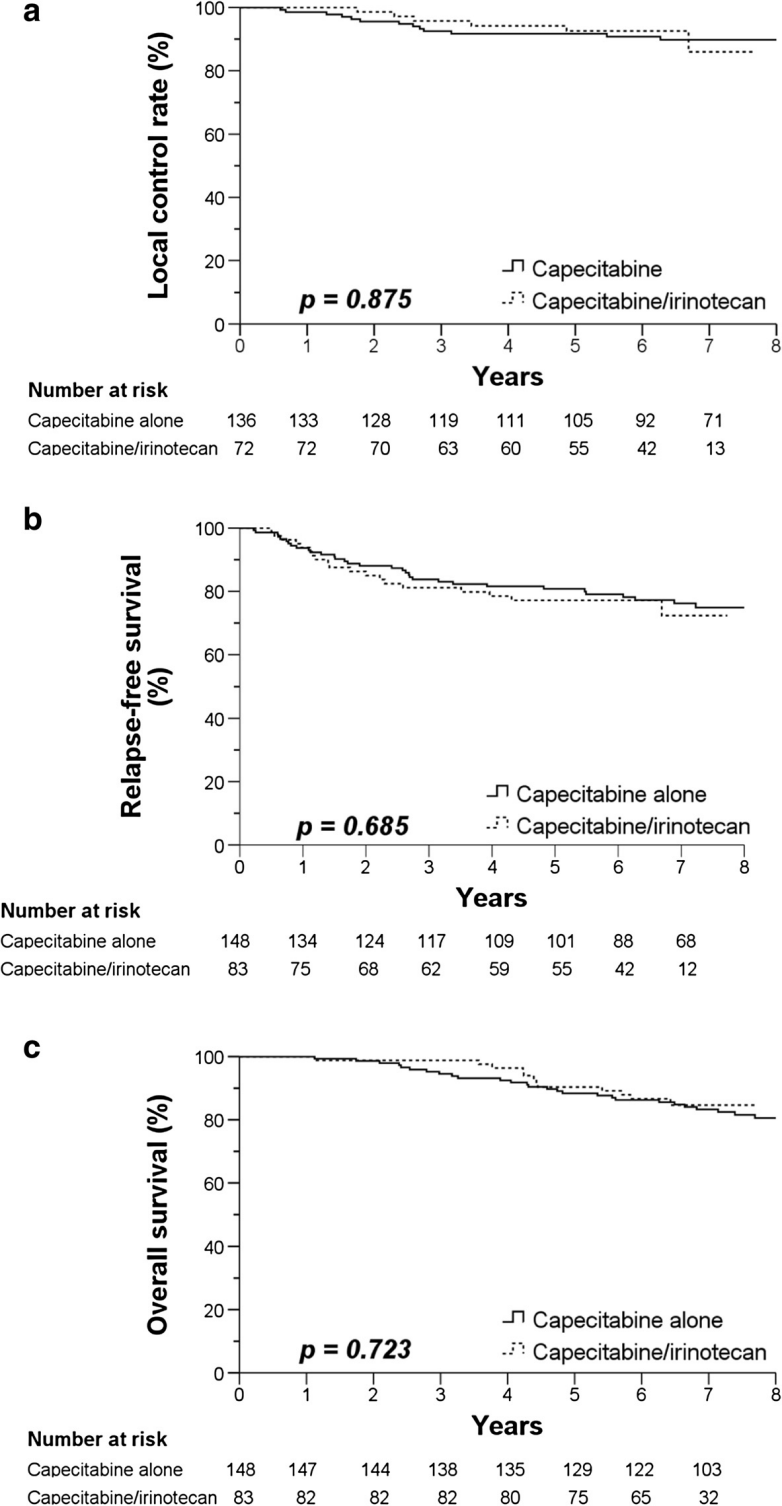
**Kaplan Meier curves of (a) Local control rate, (b) relapse-free survival, and (c) overall survival.** Solid line=capecitabine alone. Dashed line=capecitabine/irinotecan.

As of November, 2012, the median follow-up time was 77 months (range = 26–112 months). At this time point, patients in the capecitabine alone group had 5-year local control = 92.1%, relapse-free survival = 80.8%, and overall survival rates = 88.5%. In the capecitabine/irinotecan group, the patients had 5-year local control = 92.5%, relapse-free survival = 76.0%, and overall survival rates = 91.7%. No statistically significant difference was found in local control rate (*p* = 0.937), relapse-free survival (*p* = 0.484), or overall survival rate (*p* = 0.598) between the groups. Figure 1 shows the survival curve for each group.

## Discussion

Fluorouracil-based chemoradiation is one of the most widely chosen treatment options for preoperative treatment of locally advanced rectal cancer. Substituting fluorouracil with capecitabine, the fluorouracil prodrug, is attractive because of the ease of administration and mimicking of a continuous infusion [12]. A recent randomized phase III trial in Germany confirmed that the endpoint overall survival rates did not change when infused 5-fluorouracil was replaced by capecitabine during radiotherapy and adjuvant chemotherapy [4]. These findings mirror those of the large X-ACT trial [13] of adjuvant capecitabine in colon cancer, which led to FDA approval in 2005. The results of these two trials seem to warrant replacement of fluorouracil with capecitabine for adjuvant therapy of rectal cancer. This has caused increased interest in combining other chemotherapeutic agents, such as irinotecan, with capecitabine in order to enhance the effect of CRT.

Combining oxaliplatin or irinotecan with capecitabine is the most frequently attempted regimen for heightening CRT intensity because of the drug’s proven effectiveness in colorectal cancer when combined with fluorouracil [5,14]. Several randomized phase III trials adding oxaliplatin to capecitabine in preoperative CRT have been completed to determine whether this combination shows any advantage compared with the capecitabine alone. Despite the expectation of a successful outcome, early results from the NSABP R-04, ACCORD 12/0405-Prodige 2 trial did not confirm a significant improvement of short-term endpoints, such as the pathologic complete response rate, by addition of oxaliplatin [15,16]. Results of several preoperative CRT phase I/II studies using capecitabine/irinotecan at various dosages and schedules have been reported and showed encouraging tumor response rates and toxicity profiles [6,17]. Willeke *et al.*[17] reported a 15% pathologic complete remission rate and 80% 3-year overall survival rate in their phase II trial. In a capecitabine/irinotecan phase II study [7], we reported well-tolerated toxicity profiles, with a notable pathologic complete response rate (25.0%). We also reported excellent clinical outcomes with a 3-year relapse-free survival of 80.0% and overall survival rate of 94.7%. In spite of excellent survival outcomes in our phase II study, the data were interpreted cautiously due to the relatively small number of patients (*n* = 48) [7]. In the present study, 81 patients participated in the capecitabine/irinotecan regimen, leading us to expect more reliable statistical results. To assess the potential systemic benefit of irinotecan addition, it was necessary to compare long-term relapse-free survival associated with use of capecitabine/irinotecan with capecitabine alone. In the present study, the capecitabine/irinotecan regimen failed to improve relapse-free survival over capecitabine alone (capecitabine alone *vs.* capecitabine/irinotecan; 81.4% *vs.* 76.0%; *p* = 0.483). To our knowledge, this is the first study to compare directly long-term survival and short-term results in patients treated with capecitabine/irinotecan or capecitabine in preoperative CRT for rectal cancer.

In this study, we used several methods to evaluate tumor response before and after surgery. In doing so, we compared not only the early results of two regimens, but also correlated the early results with long-term clinical outcome. We estimated the tumor volume reduction rate to assess tumor response to CRT based on the recent studies which reported that it was correlated well with pathologic results and also could be a surrogate indicator of patient prognosis [18,19]. The difference in tumor volume reduction rates between the two groups was non-significant (64.8% *vs.* 64.9%; *p* = 0.990). These data therefore supported the hypothesis that the tumor volume reduction rate correlates with pathologic response and prognosis [18,19]. We also compared both regimens to determine how each regimen yielded downstaging to ypStage 0–I. Several previous studies reported that the estimation of downstaging to ypStage 0–I indicates a favorable prognostic patient group more than does assessment of the pathologic complete response (ypStage 0) and could be a reliable intermediate endpoint for preoperative CRT in rectal cancer [20]. In this context, the downstaging that occurred in each group was not significantly different (44.1% *vs.* 48.6%; *p* = 0.538), and long-term survival results were similar.

## Conclusions

This study assessed whether combining irinotecan with capecitabine was more effective in patients with locally advanced rectal cancer than capecitabine alone. Based on the radiologic and pathologic findings, we attempted to compare the short-term tumor responses of the two treatment groups. As mentioned above, we found no meaningful differences between the two groups. We also tried to compare long-term clinical outcomes of two groups by assessing the 5-year local control rate, relapse-free survival, and overall survival. Similar to the results of short-term tumor response, combining irinotecan with capecitabine did not provide any meaningful benefit in survival over capecitabine alone. Thus, the addition of irinotecan to a capecitabine regimen does not have significant advantages over capecitabine alone and is not recommended as a treatment of choice in the clinic.

## Competing interests

The authors declare that they have no competing interests.

## Author’s contributions

DYK contributed to conception and design of the study, and revised the manuscript. SUL contributed to analysis and interpretation of data, and drafted the manuscript. SYK and THK participated in revising the manuscript. HJC, MJK and JHO participated in data acquisition and literature research. JWP and JYB contributed to conception of the study. All authors read and approved the final manuscript.
